# Alternative Methotrexate Oral Formulation: Enhanced Aqueous Solubility, Bioavailability, Photostability, and Permeability

**DOI:** 10.3390/pharmaceutics14102073

**Published:** 2022-09-28

**Authors:** Bhupendra Raj Giri, Hyun Seok Yang, Im-Sook Song, Han-Gon Choi, Jung Hyun Cho, Dong Wuk Kim

**Affiliations:** 1 BK21 FOUR Community-Based Intelligent Novel Drug Discovery Education Unit, Vessel-Organ Interaction Research Center (VOICE, MRC), College of Pharmacy, Research Institute of Pharmaceutical Sciences, Kyungpook National University, Daegu 41566, Korea; 2 Pharmaceutical Engineering and 3D Printing Labs (PharmE3D), Division of Molecular Pharmaceutics and Drug Delivery, College of Pharmacy, The University of Texas at Austin, Austin, TX 78705, USA; 3 College of Pharmacy, Institute of Pharmaceutical Science and Technology, Hanyang University, Ansan 15588, Korea

**Keywords:** inclusion complex, methotrexate, solubility, bioavailability, permeability, β-cyclodextrin (β-CD)

## Abstract

The poor aqueous solubility and/or permeability and thereby limited bioavailability largely restricts the pharmaco-therapeutic implications of potent anticancer drugs such as methotrexate (MTX). Furthermore, MTX’s inherently unstable nature makes it difficult to develop a viable oral formulation. In this study we developed the spray-dried amorphous inclusion complexes of MTX with native β-cyclodextrin (β-CD) and its derivatives, namely HP-β-CD, M-β-CD, and DM-β-CD to enhance the aqueous solubility, photostability, permeability, and oral bioavailability of MTX in rats. Our findings show that the 1:1 stoichiometry ratio of MTX and CDs improves the aqueous solubility, stability, and pharmacokinetic profiles of the drug, the better results being obtained particularly with DM-β-CD as a host, which has a higher complexation ability with the drug compared to other β-CDs. Specifically, the pharmacokinetic analysis demonstrated 2.20- and 3.29-fold increments in AUC and Cmax, respectively, in comparison to free MTX. Even though the absorptive permeability of MTX and MTX/DM-β-CD inclusion complexes was similar, the efflux of the absorbed MTX from ICs was significantly lower compared to the free MTX (4.6- vs. 8.0-fold). Furthermore, the physicochemical characterization employing SEM, DSC, and PXRD confirmed the transformation of crystalline MTX to its amorphous state. In solution, ^1^H NMR studies revealed that MTX embedded into the DM-β-CD cavity resulting in both H-3 and H-5 chemical shifts implied the presence of intermolecular interaction between the drug and CD moiety. It was, therefore, evident that an MTX IC could be a successful oral formulation technique, preventing MTX degradation and enhancing its pharmacologically relevant properties.

## 1. Introduction

Methotrexate (MTX) is a potent chemotherapeutic drug whose antimetabolite activity is due to its ability to disrupt the normal mitotic cell division cycle by inhibiting dihydro-folate reductase, an enzyme involved in metabolic reactions of folic acid derivatives, thereby preventing the synthesis of DNA, RNA, and proteins [1,2]. Despite the introduction of a slew of modern cancer chemotherapies, MTX remains a common treatment option for numerous solid tumors in the head, neck, and breast, osteosarcoma, autoimmune diseases such as psoriasis, rheumatoid arthritis, and systemic inflammatory diseases [3]. Although MTX is a potent antifolate cytotoxic molecule, it belongs to a class IV molecule in Biopharmaceutical Classification System (BCS) due to its low permeability (C logP  =  0.53) and poor aqueous solubility (0.01 mg/mL at 20 °C), which results in poor bioavailability (~18% for doses >40 mg/m^2^), and thereby reduces its clinical efficacy [4]. Furthermore, it is found to be chemically unstable, decomposing quickly when exposed to light and extremes of pH or temperature [5,6]. As a result, most clinicians prefer high doses of MTX (≥500 mg/m^2^) to be administered intravenously (I.V.) in regular cycles for central nervous system (CNS) prophylaxis and osteosarcoma therapy [7]. However, this mode of drug delivery is usually painful and inconvenient to patients, and therefore results in poor patient compliance. Unfortunately, owing to the use of high I.V. doses, side effects mainly related to gastrointestinal (GI) and hepatic toxicity, stomatitis, and bone marrow suppression, among others, are sometimes fatal [8]. Therefore, the fabrication of an appropriate oral formulation of MTX that can minimize dose-related toxicity and reduce adverse effects through improved oral bioavailability and stability, and thus enhance patient compliance is imperative.

Various physical and chemical formulation approaches have been studied to enhance the solubility and bioavailability of BCS class II and IV compounds such as the modification of crystal habit [9], drug dispersion within carriers [10], lipid-based formulations [11], salt and prodrug formulations [12], particle size reduction (micronization/nanonization), and many others [13]. In this context, cyclodextrins (CDs) were identified as safe and promising pharmaceutical excipients with significant potential for drug delivery, primarily via the formation of drug-CD based delivery system [14]. CDs are macrocyclic oligosaccharides made up of six, seven or eight D-(+) glucopyranose units (α, β, and γ-cyclodextrin) connected by glucosidic bonds α(1⟶4) [15]. Because of their unique truncated cone-like configuration, which includes a hydrophilic outer shell and a hydrophobic cavity lined with protons, they allow the formation of ‘host-guest’ inclusion complexes (ICs) with a wide range of lipophilic molecules [16]. The host-guest interactions are a subset of supramolecular interactions marked by the complexation of a guest molecule with a macrocyclic cavitand (host molecules like CDs) via hydrophobic interaction, mostly enabled by van der Waal’s or electrostatic forces. These interactions are especially advantageous for encasing poorly water-soluble drug molecules, stabilizing biologically active compounds, or modulating the immune response [17]. They are capable of modulating the inherent physicochemical properties of guest molecules such as crystallinity, intrinsic aqueous solubility, permeability, and/or stability without compromising their therapeutic benefits [16]. Furthermore, their cavity size is suitable for hosting a large variety of drugs and are readily available. CD molecules are therefore being increasingly used as drug delivery vehicles and excipients in myriad pharmaceutical formulations, including oral, parenteral, ocular, nasal, pulmonary, and cosmetics (topical) [16,18,19]. Several native and chemically modified CD derivatives are being synthesized (α- β- and γ-CDs) based on the degree of methyl and hydroxy group substitution, which lends them unique physicochemical properties and inclusion complexation capability [20]. Consequently, the degree of complexation, whether in-vitro or in-vivo, correlate with the types of CDs being used in the formulation and their degree of interaction with the guest molecule. Amongst them, β-CD is the preferred and commonly used carrier in pharmaceutical formulation, largely due to its biocompatibility and biodegradability, adequate internal cavity size that can form stable IC with a large number of drug molecules, and cost advantages [21]. Furthermore, it is non-toxic and biodegradable. However, the aqueous solubility of native β-CD is limited [22], and therefore several β-CD derivatives have been synthesized such as 2-hydroxylpropyl-β-CD (HP-β-CD), methyl-β-cyclodextrin (M-β-CD), and heptakis (2,6-di-O-methyl)-β-cyclodextrin (DM-β-CD) to extend its physiochemical characteristics such as solubility, physical stability, parenteral toxicity, and complexation degree, which are of interest to our study.

In this work, we investigated the complexation of MTX with native β-CD and its derivatives for improving the pharmacologically relevant attributes i.e., aqueous solubility, photostability, apparent permeability, and bioavailability of MTX. The main objectives were to: (i) investigate the degree of complexation of various β-CD derivatives with MTX; (ii) prepare and compare the developed MTX’s ICs for solubility, bioavailability and stability advantages, and (iii) characterize and comprehend the physicochemical properties such as morphology, particle size distribution, crystallinity, and interactions between MTX and CD moiety. Prior findings have shown that complexation with CDs and their derivatives increases the biopharmaceutical performance of anticancer drugs such as docetaxel, paclitaxel, doxorubicin, curcumin, and cisplatin [23,24]. However, we found that no attempt has yet been made to assess the stability and in-vitro/in-vivo performance of MTX in presence of CD moieties. In this regard, MTX’s ICs with various β-CD derivatives were developed employing the spray-drying technique and then characterized by SEM, DSC, XRD, FT-IR, and ^1^H NMR. In addition, the complexes were compared in terms of their complexation abilities, solubility, dissolution enhancement, and increase in oral bioavailability, as well as toxicity and in-vitro permeation through Caco-2 cells, were also investigated.

## 2. Material and Methods

### 2.1. Materials

MTX (≥99%) was supplied by Huzhou Zhanwang Pharm. Co., Ltd. (Huzhou, China). The β-CD, HP-β-CD, M-β-CD, and DM-β-CD were obtained from Acros Organics (Fair Lawn, NJ, USA). All other chemicals and solvents were of reagent grade and were used as received.

### 2.2. Phase Solubility Studies

The degree of complexation between MTX and β-CDs was determined through phase solubility experiments, as established by Higuchi and Conners [25]. Briefly, an excess of MTX was added to the 10 mL aqueous solution of increasing concentrations (0 to 15 mM) containing each of β-CD, HP-β-CD, M-β-CD, and DM-β-CD. The samples tubes were then vortexed for a few seconds and placed in a water bath maintained at 100 rpm in 25 °C for 5 days (Maxturdy-30; Daihan Scientific, Wonju, Korea) to obtain the saturated complex suspension. The suspension samples obtained were then centrifuged at 10,000 rpm for 10 min and the supernatant was collected, and filtered using a 0.45 µm syringe filter. The samples were then diluted with a mobile phase to obtain the suitable concentrations before being quantified using a HPLC system. All concentrations were made in triplicate (*n* = 3).

The outcomes of the phase solubility tests were plotted against the known *CD* and MTX concentration in the sample and the resulting regression profiles was used to derive the association constant (*K_α_*). The apparent association constant (*K_α_*) was calculated from the phase solubility curve according to the following equation:

(1) $$K_{\alpha }=\frac{\left[D\;:CD\right]}{\left[D\right]\times \left[CD\right]}=\frac{Slope}{S_{o}\left(1-Slope\right)}$$

In the case of very poorly soluble drugs, the *Slope* is the linear part of the plotted curve and *S_o_* is the inherent solubility of the associated drug at 25 °C, also extrapolated as the intercept from the linear equation.

The complexation efficiency (*CE*) of the IC system was calculated from the *Slope* of the phase solubility profiles.

(2) $$CE=\frac{\left[D\;:CD\right]}{\left[CD\right]}=S_{o}\times K_{\alpha }=\frac{Slope}{\left(1-Slope\right)}$$

### 2.3. HPLC System

Quantification of MTX in the samples was conducted using a HPLC system and the methods were the same as those used in previous studies [26]. The HPLC system was equipped with an Agilent 1260 VWD detector, an Agilent ChemStation software (version B.04.02), and an Inertsil ODS-4 C18, 250 × 4.6 mm (5 μm) column maintained at 25 °C. The mobile phase was composed of a mixture of methanol and 0.1 M dibasic phosphate (pH adjusted to 3.0 with HCl solution) in a ratio of 26:74 *v*/*v*, respectively. The UV spectrum was recorded at 303 nm with a flow rate of 1 mL/min and an injection volume of 10 µL. 

### 2.4. Preparation of the Binary Inclusion Complex and Solubilization Test

Spray-dried MTX-CD ICs were prepared in a 1:1 molar ratio with β-CD and its derivatives at different compositions (Table 1). Briefly explained, the calculated amount of CDs was dissolved in 200 mL of Milli-Q water, followed by the addition of 200 mL of MTX in 0.01 M HCl ethanolic solution. The mixture was stirred at room temperature for 24 h, allowing the formation of ICs in the solution state. For the removal of the solvent, the resulting clear solution was spray-dried using a laboratory-scale spray dryer (Büchi B-290; Büchi Co., Flawil, Switzerland). For spray drying, the inlet temperature was set at 110 °C with an outlet 75–85 °C, respectively; feed rate of 3 mL/min; atomization pressure 0.15 MPa, and aspiration was 100%. The resulting spray-dried powders were scraped off the collector and stored in an amber color glass for protection from light until further use. The aqueous solubility of all the spray-dried MTX-CD ICs powder was performed according to the phase solubility study, as described earlier. All the studies were performed in triplicate (*n* = 3).

### 2.5. In Vitro Dissolution Study

In vitro dissolution profiles of free MTX and MTX-CD ICs powder (equivalent to 50 mg of MTX) were carried out using the USP type II, dissolution apparatus (DT 620; ERWEKA, Langen, Germany). The powder samples were manually filled into capsules (size 0) and placed into the dissolution media consisting of 900 mL of Milli-Q water (pH 7.2) at 37 ± 0.5 °C, at a stirring speed of 100 rpm. At different time intervals (0, 5, 10, 20, 30, 45, and 60 min), 1 mL of aliquot was drawn and replaced with an equal volume of fresh media. After the dissolution testing, the collected samples were filtered using a syringe filter (PTFE 0.45 μm) and then the quantification of the drug in the samples was determined by the HPLC, as described above. The tests were performed in triplicate (*n* = 3) and the results are reported as mean ± Std. deviations.

### 2.6. The Physicochemical and Morphological Characterization

#### 2.6.1. Particle Size and Morphology Analysis

The complete particle size distribution of free MTX powder and all four MTX-CD IC formulations was investigated by laser diffraction (Mastersizer 3000; Malvern Instruments, Malvern, UK). The particle size/distribution is expressed as Dx10, Dx50, and Dx90, taken from the cumulative distribution, where the particle diameters are measured at the 10th, 50th, and 90th percentiles, respectively. The experiments were performed in triplicate.

A scanning electron microscopy (S-4800; Hitachi, Tokyo, Japan) was used to examine the shape and surface morphology of MTX, β-CD and its derivatives, and all four MTX-CD IC formulations. The powder samples were fixed onto a brass stub with double-sided adhesive black tape and made electronically conductive by platinum coating (6 nm/min) under vacuum using a Hitachi Ion Sputter (E-1030) at 15 mA for 120 s. Microscopic images were then taken from multiple spots.

#### 2.6.2. Differential Scanning Calorimetry (DSC) and Powder X-ray Diffraction (PXRD)

Thermal characteristics of the powder samples were carried out using a DSC (Q20; TA Instruments, New Castle, DE, USA). Samples of 5−10 mg were weighed and sealed in an aluminum pan. An empty pan was used as the reference, and the baseline, temperature, and enthalpy were adjusted. The samples were scanned at 10 °C/min in the temperature range of 50 °C to 180 °C with a 50 mL/min nitrogen flow rate for each DSC run.

The crystallization characterization of the samples was then assessed using an Empyrean multi-purpose XRD (D/MAX-2500; Rigaku, Tokyo, Japan) with Cu-Kα radiation (λ = 1.54178 Å), a voltage of 40 kV, and current intensity of 40 mA. A glass sample holder was mounted with the sample powder and the XRD curves were obtained between 5° and 45° (2θ diffraction angle) with a step scan mode of 0.05°/s and scanning speed of 3°/min at room temperature. 

#### 2.6.3. Fourier-Transform Infrared Spectroscopy (FTIR)

The powdered samples (MTX, four CDs, PMs, and ICs) were characterized using a FTIR spectrometer (FTIR-4100; JASCO, Easton, MD, USA) equipped with a diamond attenuated total reflectance (ATR) component. The samples were scanned over the spectral region of 4000–400 cm^−1^. With the FTIR spectroscopy studies, we assessed the changes in the peak shape, peak positions, and intensities and thereby confirmed the formation and interactions of IC.

#### 2.6.4. ^1^H NMR Studies

The ^1^H NMR spectra and 2D NOESY spectra of MTX, DM-β-CD, and IC4 formulation were recorded at 500 MHz on a Bruker DRX 500 NMR spectroscopy (Bruker Advance, Rheinstetten, Germany) at 25 °C. The powder samples were diluted to ~10 mg/mL solution in deuterated dimethyl sulfoxide-d6 (DMSO-d6) and transferred to NMR tubes (ϕ = 5 mm) for analysis. The chemical shift changes (Δδ) were reported in ppm employing tetramethylsilane (TMS) as an external standard. 

### 2.7. In Vivo Pharmacokinetic Studies 

To assess the in vivo pharmacokinetic parameters of free MTX and MTX-CD IC formulations, 30 male Sprague-Dawley rats (6–8 weeks old, weighing 280 ± 20 g) were used (Nara Biotech., Seoul, Korea). All the animals were fed with a normal laboratory diet and water and left for acclimatization in a laboratory environment for 3 days at 23–25 °C/50–55% RH. Care and handling of animals complied with the provisions of guidelines defined and agreed upon by the Institutional Animal Care and Use Committee at the Kyungpook National University (permit number. 2019-0054).

Before the experiment, the animals were divided into five groups with 6 animals per group and made to fast overnight. Each rat, anesthetized with ether, was fixed on a surgical board in the supine position by a thread. A polyethylene tube containing 50 IU/mL of heparin in saline was inserted into the right femoral artery of the rat for cannulation. Then, either 1 mL aqueous suspension of free MTX or the four MTX-CD IC formulations (Equiv. to 20 mg/kg of MTX) was administered orally to each of the rats using oral gavage. Blood samples (~0.3 mL) were drawn from the planted tube at various time periods of 5, 15, 30, 60, 120, 240, 480, 720, and 1440 min after the oral administration. The samples were then immediately transferred into heparin-containing microtubes and centrifuged at 10,000 rpm for 10 min to extract the plasma. The extracted plasma samples were transferred to other microtubes and stored at −20 °C until further analysis by HPLC.

#### Sample Preparation and Chromatographic Analysis

Plasma (90 μL) was mixed with 10 μL of theophylline solution (1000 μg/mL in MeOH) as the internal standard and 100 μL of methanol, respectively. The samples (200 μL) were then centrifuged at 13,000 rpm for 10 min and the upper clear supernatant layer was drawn and injected into the HPLC system (20 μL) for the quantification of MTX. In addition, the blood samples drawn at pre-dose (0 h) were used to draw a six-point calibration plot. The *Slope* of the graph was linear with R^2^ = 0.995 over the range of 25–2000 ng/mL. The mobile phase composition, flow rate, analytical column used, and UV wavelength of the HPLC method was similar to that described in Section 2.3. The pharmacokinetic (PK) parameters such as maximal plasma concentration (C_max_), time to reach C_max_ (T_max_), were obtained from the plasma concentration vs. time plots. Moreover, the area under the plasma concentration vs. time curve (AUC_0–∞_) was calculated using the linear trapezoidal summation rule and elimination half-life (t_1/2_), and elimination rate constant (K_el_) were obtained using non-compartmental model WinNonlin software (Pharsight Corp., Mountain View, CA, USA). Differences in the PK parameters were assessed with the one-way ANOVA test and a *p* value of less than 0.05 (*p* < 0.05) was considered statistically significant.

### 2.8. Photostability Assessments

The free MTX powder and the prepared IC formulations were subjected to UV irradiation for a period of 20 days. The powdered samples were spread over the bottom surface of transparent glass vials and stored at 25 °C/60% RH in a photostability chamber (Model 6545-2; Caron, Marietta, OH, USA). There, the samples were exposed to a light source of 1.2 million lux·hr intensity. At scheduled time intervals, a few mg of the sample was drawn, and assayed for the drug content by HPLC method, as described above. At the end of the study period, the samples were physically inspected for any physical changes on the stability of MTX.

### 2.9. Permeation Experiments through Caco-2 Cells

#### 2.9.1. In Vitro Cytotoxicity Study

An MTT assay was used to assess the potential cytotoxic behavior of MTX and MTX/DM-β-CD on human intestinal epithelial cancer cells (Caco-2). The Caco-2 cells were grown in DMEM supplemented with 10% FBS, 1% *v*/*v* sodium pyruvate, L-glutamine, penicillin, and MEM non-essential amino acids. The cells (2 × 10^4^ cells/well) were then seeded onto 96-well transwell plates and incubated at 37 °C with 5% CO_2_ for 24 h. New medium containing the tested formulations i.e., MTX and MTX/DM-β-CD (IC4), with varying concentrations, were added and incubated for 24 h. The culture medium was then aspirated, and the viability of the cells was determined by adding 20 μL/well of MTT solution to each 96-well plate and incubating for another 4 h. The formazan crystals were dissolved by adding 150 μL/well DMSO. The developed formazan was assayed at 570 nm, using a microplate reader with untreated cells serving as a control. All the tests were carried out in two microplates with six parallel wells each.

#### 2.9.2. In Vitro Caco-2 Permeation Experiment

The in vitro permeability of MTX and MTX/DM-β-CD formulation was evaluated using Caco-2 cells (passage no 45) and performed like the previously reported method [27]. The cells were seeded (5 × 10^5^ cells/well) onto filter inserts from 12-well transwell plates and grown at 37 °C with 5% CO_2_ for 24 h. The cells were then cultured for three weeks, with the media being replaced on alternate days. Caco-2 cells with TEER ≥ 500 Ω·cm^2^ were used in this experiment, which is indicative of adequate monolayer formation. 

To evaluate the A to B transfer, the medium from each well was replaced with prewarmed HBSS (washing of monolayer) and maintained at 37 °C for 15 min. The HBSS solution on both sides of the cell monolayer was removed by decanting and aspiration from the lower end of the plate well. Then, 0.5 mL of HBSS (pH 7.4) medium containing 50 µg/mL of MTX alone or eqvt. concentration of MTX/DM-β-CD formulation was placed to the dosing compartment (A) and 1.5 mL of fresh HBSS in the receiving compartment (B). Similarly, efflux i.e., B to A transport of MTX and MTX/DM-β-CD was carried out, but the dosing and receiving side were changed to B and A, respectively. After withdrawing 0.2 mL samples from the receiving compartment in every 15 min for 1 h, an equal volume of fresh HBSS was replenished to maintain a constant volume. To each collected samples (0.2 mL) an internal standard solution (1 ng/mL berberine in MeOH) was added and vortexed (15 min), followed by centrifugation at 16,000 g for 5 min. Then, the clear supernatant (5 µL) was injected into an Agilent 6430 Triple Quadrupole HPLC/MS in a 5 L aliquot (Agilent, Wilmington, DE, USA) equipped with Synergy polar RP column (2 × 150 mm, 4 µm; Phenomenex, CA, USA). The mobile phase consists of methanol and water with 0.1% formic acid (20:80 *v*/*v*) maintained at 0.2 mL/min flow rate. Electrospray ionization in the positive mode was used to record mass spectra. MTX was quantified at m/z 455.2 ⟶ 308.1 while berberine at m/z 336.1 ⟶ 320.0.

The apparent permeability coefficient (*P_app_*, cm/s) was calculated as:

(3) $$P_{app}=(dQ/dt)/(C_{0}\times A)$$

The efflux ratio (*ER*) was calculated as:

(4) $$\mathrm{ER}=P_{app\;}(B\to A)/P_{app\;}(A\to B)$$

where, $P_{app\;}$ (B → A) and $P_{app\;}$ (A → B) represents the apparent permeability of the investigated formulations.

## 3. Results and Discussion

### 3.1. Phase Solubility Studies

**Figure 1:**
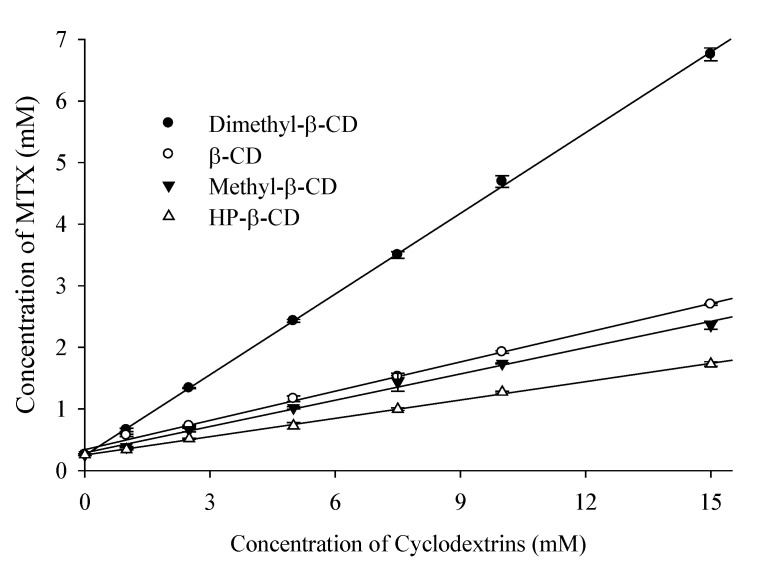
Phase solubility diagram of MTX as a function of different cyclodextrin concentrations (*n* = 3).

The study of phase solubility is one of the basic criteria to access the types of complexations formed between drug and CD moieties. Furthermore, the phase solubility plots of the drug in presence of increasing CDs concentration are useful for determining the affinity between the drug and CDs molecules. In this regard we investigated the phase solubility of MTX in presence of various pharmaceutically accepted β-CD and its derivatives, mainly HP-β-CD, M-β-CD, and DM-β-CD. As shown in the phase solubility diagram, Figure 1, the apparent aqueous solubility of MTX increases in a linear fashion as a function of the CD concentration, which represents the formation of “AL” type complexation, as described by Higuchi and Connor [25]. The graphs were found to exhibit linear host–guest correlation with R^2^ > 0.99 and *Slope* > 1, which confirms the formation of ICs with 1:1 (MTX:CD) stoichiometric ratio. In addition, DM-β-CD showed the maximum drug solubility (from 0.34 to 1.73 mM) while it was least improved in the presence of HP-β-CD (from 0.66 to 6.76 mM). 

Amongst the different CDs used, the apparent stability constant (*K_α_*) value of 3114.69 M^−1^ indicates that MTX and DM-β-CD molecules have the highest affinity for one another, therefore the MTX/DM-β-CD IC has greater solubilization efficiency, and therefore superior stability (Table 2). These differences in *K_α_* values lie in the properties of different CDs, such as height, internal cavity diameter, and volume, which largely affect the degree of complexation with the guest molecule [21]. Our prior findings as well as other studies have reported that the hydrophobic DM-β-CD cavity has a greater affinity for the lipophilic drug molecules due to the presence of methyl groups in its inner cavity, which allows enhanced flexibility and therefore, the formation of stable ICs [28,29]. On the other hand, the *K_α_* value for MTX with HP-β-CD was the least (433.56 M^−1^), which suggests that the complexation of the drug within HP-β-CD cavity was the least favorable. Lipophilic molecules, in general, have a greater affinity for the more hydrophobic CD cavity, resulting in a higher *K_α_* value. These findings support the concepts that the types of CDs used in the complexation have a significant influence on the degree of complexation between drug and CD and hence, on the stability and solubilization capacity of the developed formulation. 

### 3.2. Solubility and In-Vitro Dissolution Studies

**Figure 2:**
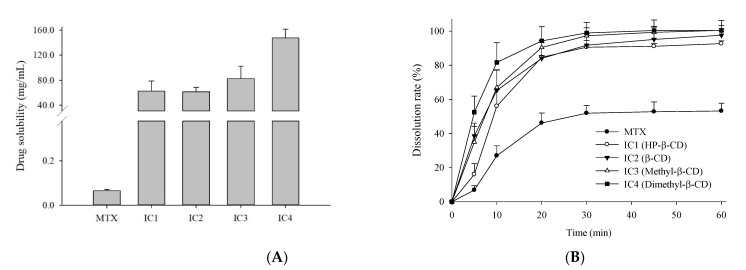
(**A**) Solubility and (**B**) dissolution profiles of MTX and the four different MTX-CD inclusion complex formulations; MTX/HP-β-CD inclusion complex (IC1); MTX/β-CD inclusion complex (IC2); MTX/M-β-CD inclusion complex (IC3); MTX/DM-β-CD inclusion complex (IC4) in aqueous media. Each value represents the mean ± SD (*n* = 3).

Four spray-dried MTX-loaded inclusion complexes i.e., MTX/HP-β-CD (IC1), MTX/β-CD (IC2), MTX/M-β-CD (IC3), and MTX/DM-β-CD (IC4) were investigated for the solubility and in vitro drug release studies. As represented in Figure 2A,B, aqueous solubility and the cumulative volume of MTX released from all four IC formulations were substantially higher than the pure drug, which presumably led to faster in vivo drug absorption in the rats. The in vitro drug release kinetics of the four ICs within 10 min were 56.0%, 65.3%, 67.0%, and 81.7%, respectively, which reached to 92.7%, 97.6%, 100.5%, and 100.4%, respectively, within 1 h. No lag phase was observed on quantifying the dissolution samples at the initial time points, indicating that the drug releases from the filled hard gelatin capsules were prompt. A burst release with maximum drug concentration was achieved within the first half of the dissolution study, following plateau curves thereafter. This burst release at the initial stage of the dissolution is presumed to be due to the prompt release of loosely encapsulated MTX molecules in the CD cavity or the existence of free solubilized amorphous drug, which is rapidly dissolved when it comes into contact with the dissolution media leading to the rapid drug release profile. This kind of drug release behavior from the CD-based IC systems has been reported in prior findings [28,30].

Overall, all the spray-dried IC formulations demonstrated enhanced solubility and dissolution of MTX compared to the free drug, the best results being obtained when using DM-β-CD as a host, due to its higher complexation ability with the MTX compared to other β-CD used in this study. In particular, the solubility and in vitro drug release of MTX from IC4 formulation was 2268-fold and 1.89-fold higher than the free MTX. These results may be attributed to the following reasons: (i) the inclusion of lipophilic drug into the hydrophobic cavity of the native CD moiety, and thus the formation of IC with higher solubilization ability, leading to increased dissolution; (ii) the amorphous transformation of crystalline drug renders higher enthalpy, entropy and free energy than its crystalline form; and lastly, (iii) the reduced particle size during processing and thus, the large surface area available for dissolution accelerated and improved the drug dissolution [31,32]. Hence, CDs complexation of MTX molecule induced a significant increase in solubility and dissolution.

### 3.3. Physicochemical Characterization

#### 3.3.1. SEM Analysis

**Figure 3:**
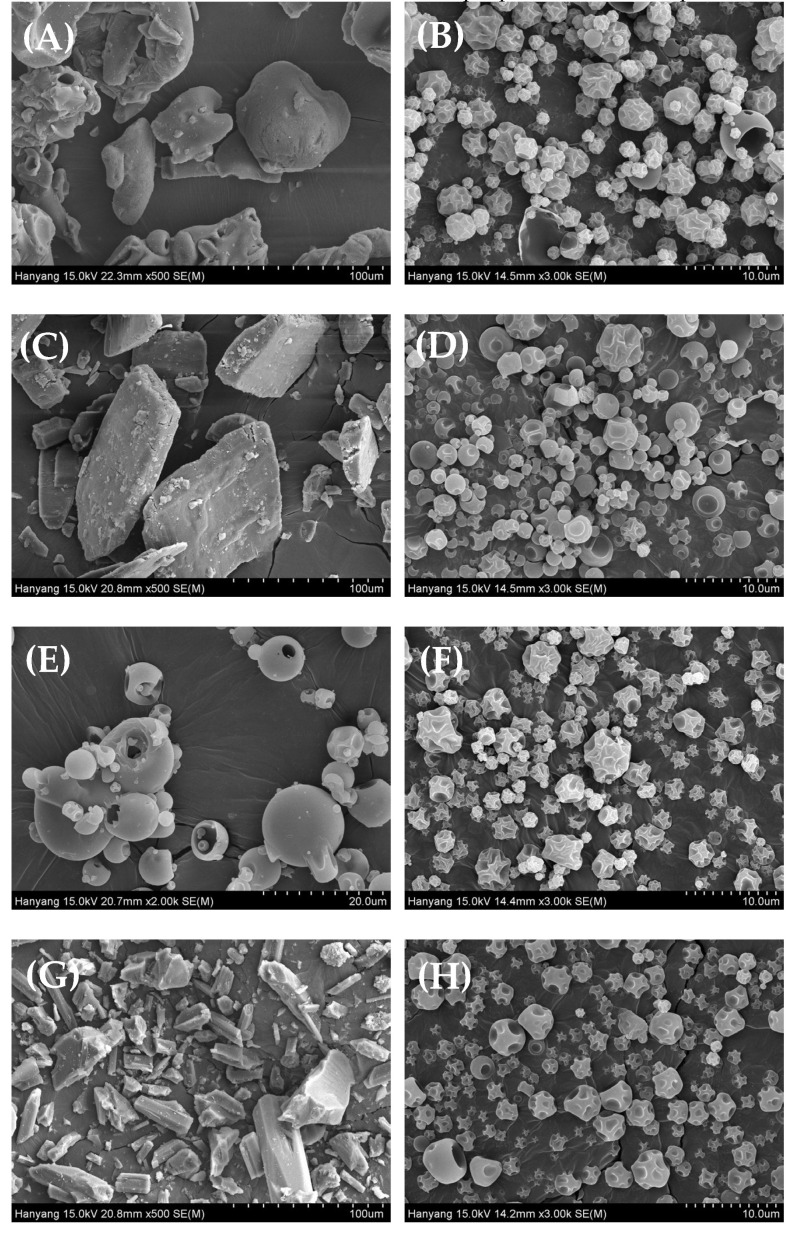
Scanning electron micrographs: (**A**) HP-β-CD (×300); (**B**) MTX/HP-β-CD inclusion complex (IC1) (×3000); (**C**) β-CD (×500); (**D**) MTX/β-CD inclusion complex (IC2) (×2000); (**E**) Methyl-β-CD (×2000); (**F**) MTX/M-β-CD inclusion complex (IC3) (×2000); (**G**) Dimethyl-β-CD (×500); and (**H**) MTX/DM-β-CD inclusion complex IC4 (×3000).

SEM was used to examine the surface morphology and roughly estimate the particle size of free powder samples as well as the changes that occurred after spray drying of ICs. Figure 3 shows the differences between the SEM micrographs of the free MTX powder and the four IC formulations. It can be seen in the images that free drug powders are characteristic compact particles with sharp edges and crystal-shape (Figure 3A). Furthermore, the morphology of CDs varies. M-β-CDs are sphere-shaped, slightly smooth surface particles while the rest of the CDs (HP-β-CD, β-CD, and DM-β-CD) have bulky, bar-like shapes with a rough outer appearance. In contrast, all four IC formulations appeared to have surface morphology and size compared to the drug and CD molecules. In particular, the ICs have a smooth texture and well-defined sphere-shaped particles with a few dents on their surface. These dents are typically generated during the spray drying process and observed in many cases with the spray-dried particles, which might be due to rapid solvent evaporation from the surface of the micro-nano sized spraying particles when it comes into contact with the drying gas maintained at a high temperature [33,34]. These changes in the morphology can be taken as supportive evidence of the complexation between the guest and host in the IC [35]. In addition, through the SEM images and particle size evaluation, reduced and uniform particle size of ICs was confirmed (Table 3). Further, DSC and XRD were used to assess the solid-state properties of the samples in more detail.

#### 3.3.2. Differential Scanning Calorimetry (DSC)

**Figure 4:**
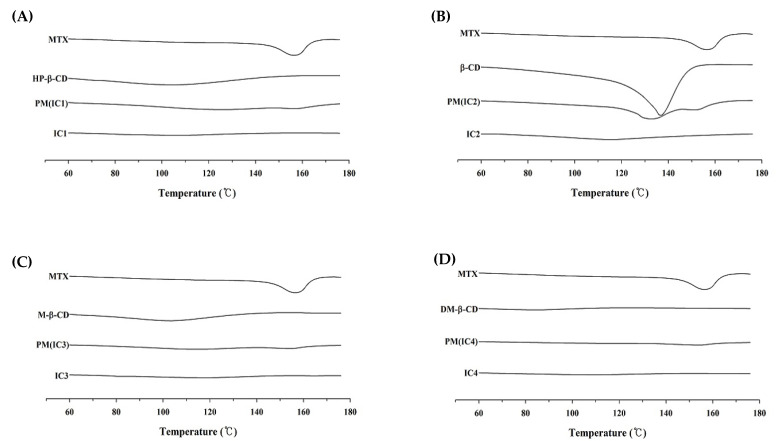
DSC thermograms of the MTX powder, cyclodextrins, physical mixtures, and MTX-CD inclusion complex formulations: (**A**) MTX/HP-β-CD inclusion complex (IC1); (**B**) MTX/β-CD inclusion complex (IC2); (**C**) MTX/M-β-CD inclusion complex (IC3); and (**D**) MTX/DM-β-CD inclusion complex (IC4).

The DSC curves of free MTX, CDs and all four ICs are shown in Figure 4. The DSC thermogram of MTX (Figure 4A) shows a small, broad endothermic peak at 150–165 °C, which corresponds to the melting temperature of MTX and confirms its crystalline nature. The thermal curves of all the CDs, including HP-β-CD, M-β-CD, and DM-β-CD, are flat shaped, while native β-CD has a large and broad endothermic peak ranging between 120–150 °C, which might be due to dehydration (liberation of crystal water) of β-CD [36,37]. The characteristic endothermic peak of MTX was absent across the entire scanned temperature range in the DSC thermogram of ICs, however. This is an indication that MTX may have translated from its crystalline form to an amorphous state because of the higher affinity of solid-solid interactions following complexation. Usually, when a drug molecule is complexed with CD moiety, the drug peak shifts to a lower temperature or disappears entirely, indicating a loss of crystallinity in the drug molecule. In addition, significant crystallinity loss of MTX can be observed with the diffraction patterns of all PMs, which might be due to the dilution effect or physical dispersion of the drug within the drug-carrier mixture [38]. Furthermore, because DSC has a limited resolution [39], it may not have spotted MTX crystallinity in the PM, thus we used XRD to further characterize these samples.

#### 3.3.3. Powder X-ray Diffraction (PXRD)

**Figure 5:**
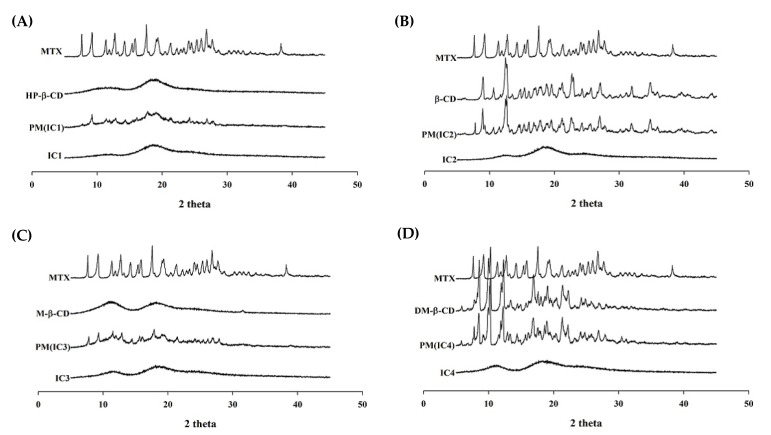
PXRD patterns of the MTX powder, cyclodextrins, physical mixtures, and MTX-CD inclusion complex formulations: (**A**) MTX/HP-β-CD inclusion complex (IC1); (**B**) MTX/β-CD inclusion complex (IC2); (**C**) MTX/M-β-CD inclusion complex (IC3); and (**D**) MTX/DM-β-CD inclusion complex (IC4).

We studied the XRD patterns of the drug, CDs, their PMs, and all four ICs to see if the drug had undergone any solid-state conversion from its original lower energy crystalline form to a much higher energy amorphous state, and the findings are depicted in Figure 5 for visual comparison. The appearance of several characteristic sharp and large diffraction peaks in the diffractogram of MTX suggests that the drug exists in its inherent crystalline state. Similarly, the presence of distinguished sharp peaks of MTX in the thermogram of the PMs implies that the drug is still in its crystalline form. The peak intensities in PMs were found to be slightly diminished, which could be the result of the dilution and/or partial interaction of MTX with CD molecules [38]. Also, the presence of a drug peak, even if it is faint, suggests that the IC has not formed completely. All four ICs diffractograms, on the other hand, lacked any apparent sharp diffraction peaks rather displaying a halo pattern, indicating complete drug amorphization, which is attributed to the supramolecular interactions between MTX and CD, and thereby inclusion of the drug in the CD cavity. Based on the SEM, DSC, and XRD observations, we can surmise that MTX when embedded in the CDs cavity results in the formation of binary ICs, thereby transforming from its native crystalline state to amorphous nature. The findings are consistent with existing research findings, which suggest an absence of the drug’s crystallinity in the IC formulations is attributed to true complexations between the drug and CD moiety [40,41]. This transition from crystalline to amorphous form is largely beneficial as higher enthalpy and entropy of amorphous form results in higher molecular kinetics, as a result, a higher degree of drug solubilization can be achieved. Based on solubility, in-vitro dissolution, in-vivo pharmacokinetics, and photostability data, it is apparent that the MTX/DM-β-CD (IC4) is a more suitable formulation with better overall biopharmaceutical profiles than the rest of the MTX-IC formulations, therefore, we decided to choose IC4 formulation for our further studies.

#### 3.3.4. FT-IR Spectroscopy

**Figure 6:**
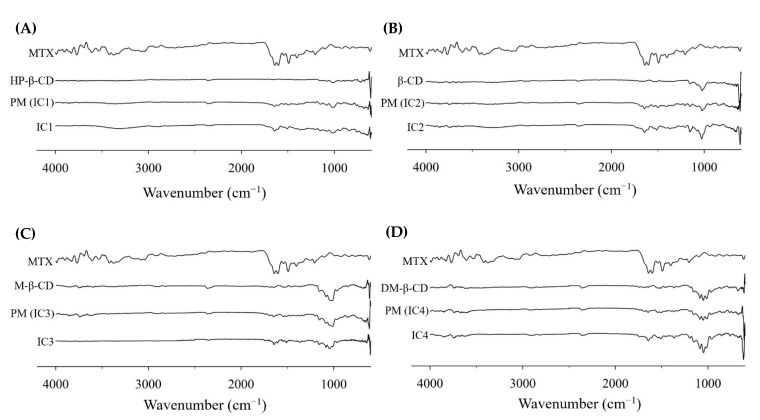
FTIR spectra of the MTX powder, cyclodextrins, physical mixtures, and MTX-CD inclusion complex formulations: (**A**) MTX/HP-β-CD inclusion complex (IC1); (**B**) MTX/β-CD inclusion complex (IC2); (**C**) MTX/M-β-CD inclusion complex (IC3); and (**D**) MTX/DM-β-CD inclusion complex (IC4).

The DSC and XRD data only offer basic insights into the supramolecular structure and no unequivocal evidence on the inclusion of MTX in CDs, thus the FT-IR and ^1^H NMR were used to gain a better understanding of the molecular conformation of ICs. The change in IR spectrum of the guest molecule before and after complexation, i.e., shifting to higher or lower wave number, complete peak masking, and/or discrepancies in IR spectrum intensity, provides a general understanding of host–guest interactions between drug and CD [42,43]. Figure 6 shows the IR spectrum of samples (MTX, four CDs, PMs, and ICs) at 400–4000 cm^−1^ wavenumber.

The characteristic peaks for DM-β-CD were recognizable by a broad and large band between 3500 and 3300 cm^–1^ due to intermolecular–OH bonding, 2921 cm^–1^ –CH and –CH_2_ stretching vibration, 1451 cm^–1^ is due to H–O–H bending vibration, and between 1200 and 1040 cm^−1^ reflects the –C–C– vibrations, C–N and C–O stretching modes [28]. The typical absorption bands of MTX and DM-β-CD in the PM are almost identical with a simple overlap of MTX and DM-*β*-CD IR spectrum, indicating that there was no apparent interaction between the drug and CD. On the other hand, the IR spectrum of MTX in IC4 (Figure 6D) showed a marginal shift in the absorption bands of MTX towards a higher frequency with a lower intensity. Moreover, the characteristic sharp peaks of MTX at 3352 cm^−1^ (stretching of N–H), split band between 1600 and1500 cm^−1^ (C=C stretching from the carboxylic group and amidic group) were largely weakened and the peak near 1450 cm^−1^ (C–H) was completely masked. These changes in MTX’s IR spectrum could be ascribed to the dissociation of intermolecular H bonds of MTX because of complexation with DM-*β*-CD, indicating the formation of MTX/DM-*β*-CD ICs. These findings agree with the prior reports in which the loss in absorption bands intensity and/or peak masking of the drug molecule was accounted for the host–guest interactions between drug and CD [44,45].

#### 3.3.5. ^1^H NMR and 2D NMR Studies

**Figure 7:**
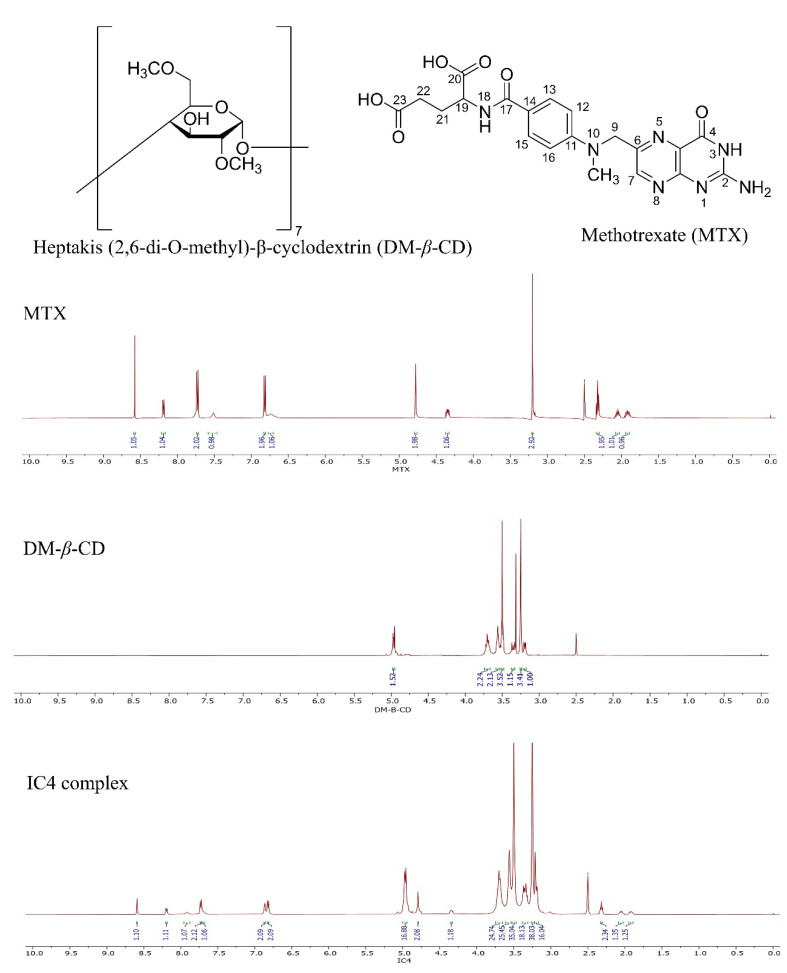
^1^H NMR spectra of MTX; DM-β-CD; and MTX/DM-β-CD (IC4).

The potential inclusion mode of the MTX/DM-*β*-CD was investigated using ^1^H NMR spectroscopy. The protons in the chemical and electronic environments were altered because of the interactions between the guest and host molecules, as evidenced by NMR signals and chemical shift values (Δδ). Hence, the changes in Δδ support the formation of IC and larger chemical shifts are associated with more fruitful drug-CD interactions [45]. In this study, the ^1^H NMR chemical shifts (δ ppm) corresponding to protons of MTX before (δ*_free_*) and after the formation of a complex with DM-β-CD (δ*_complex_*), as well as changes (Δδ ppm = δ*_complex_* − δ*_free_*), are depicted in Figure 7 and listed in Table 4. 

From the Table 4, it is apparent that the chemical shift (Δδ) values of H-1, H-2, and H-4 protons which lies on the outer surface of the cone-like DM-*β*-CD were relatively unaffected before and after the complexation compared to H-3 (3.20–3.26 ppm, δΔ = 0.06 ppm, downfield) and H-5 (3.34–3.36 ppm, δΔ = -0.02 ppm, upfield), which implies that the H-3 and H-5 of DM-*β*-CD were involved in the complexation. If a guest molecule is embedded into the CD (host) cavity, the protons in the cavity (H-3 and H-5) are sensitive to the changing environment and experience a greater shielding/de-shielding effect with the formation of ICs, consequently higher chemical shift values than those of outer surface protons (H-1, H-2, and H-4) [28,46]. The dense electronic clouds at the H-5 of the DM-β-CD made it be shielded and upfield shift, while the H-3 protons presented downfield shift owing to the de-shielding effects of the Van Der Waals interaction between DM-*β*-CD and guest molecules or local polarity change after complexation [45].

Concurrently, the inclusion pattern of the MTX/DM-*β*-CD was explored further by examining the ^1^H NMR spectrum of MTX with or without DM-*β*-CD. As illustrated in Figure 7, some of the MTX signals appeared at 1.92–4.78 ppm, which were presumably similar to the DM-*β*-CD protons (3.20–4.97 ppm). As a result, many MTX proton signals overlapped in the spectra of the IC, making them difficult to distinguish. Due to the lower percentage of MTX in the IC, MTX proton signals were weakened than DM-*β*-CD. Furthermore, from Table 4, we can observe that DM-*β*-CD mediated chemical shift differences existed mainly in the protons in positions, -NH_2_ (4) (δΔ = 0.39 ppm), -NH_2_ (2) (δΔ = 0.13 ppm), H-22 (δΔ = 0.02 ppm), H-21 (δΔ = 0.01 ppm), H-7 (δΔ = 0.02 ppm), and H-9 (δΔ = 0.01 ppm). These findings suggest that the -NH_2_ group at 2, 4 positions and -C-H group at 21, 22 positions are most likely to enter the DM-*β*-CD cavity and interact via intermolecular hydrogen bond to form the MTX/DM-*β*-CD inclusion complex. This is also in accordance with the FTIR result since the peak loss and loss in the IR band spectrum of IC4 occurs at this position. The H-5 protons are near the narrow side of the DM-*β*-CD cavity, while H-3 protons are near the broad side [15]. When the complex was formed, the drug molecule enters the DM-*β*-CD cavity from the wider side since, Δδ value of H-3 > H-5 and penetrates into the DM-*β*-CD as a result, both the H-3 and H-5 are perturbed substantially by the MTX.

**Figure 8:**
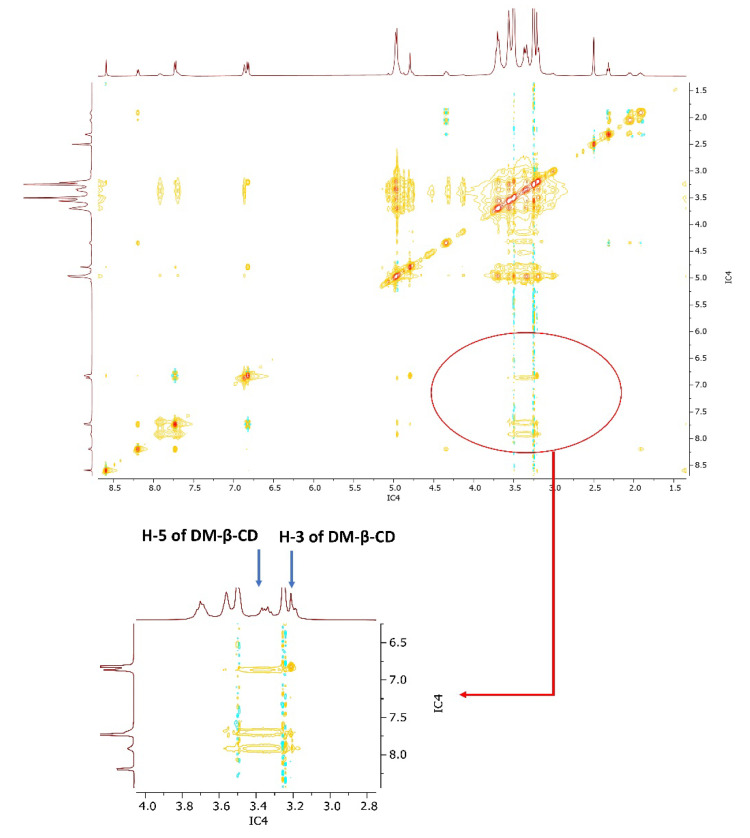
Two-dimensional (2D) NOESY spectrum of MTX/DM-β-CD inclusion complex (IC4) in DMSO.

To gain further insight into the possible inclusion pattern of MTX and DM-*β*-CD in the IC, two-dimensional (2D) NMR spectroscopy NOESY was carried out. Two protons in close proximity within 0.4 nm can produce a nuclear Overhauser effect displayed by cross-correlation in the NOESY spectrum [47]. Thus, from Figure 8, an obvious NOESY correlation between the protons of MTX and H-3 and H-5 protons of DM-*β*-CD could be found, which suggests that MTX was embedded into the DM-*β*-CD cavity from the wider side, which may be via the polar hydrogen bonding and the weak van der Waal interactions. Further investigation, including a molecular docking study, might be helpful to accurately comprehend these experimental results.

### 3.4. In Vivo Pharmacokinetic Studies

**Figure 9:**
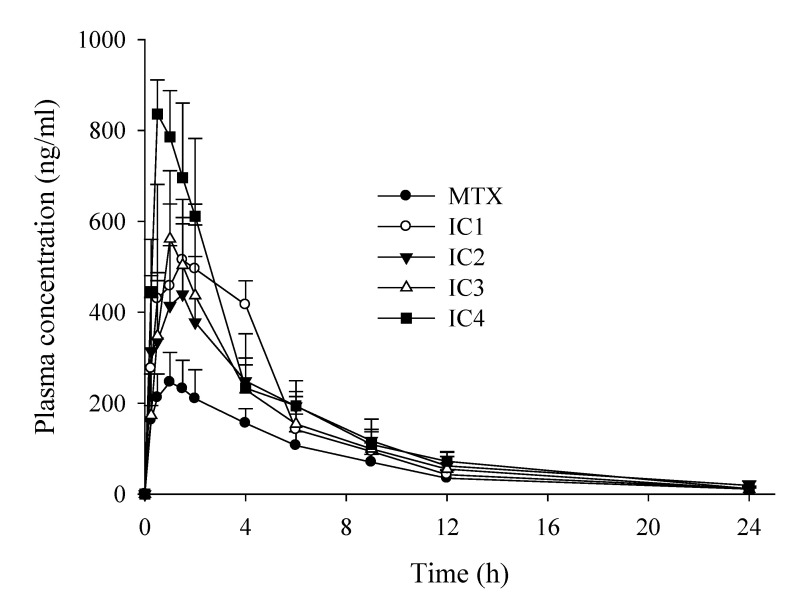
Plasma concentration–time profiles of MTX after oral administration of MTX and inclusion compounds in rats. Each value represents the mean ± S.D. (*n* = 6).

The oral bioavailability of free MTX and all four IC formulations was assessed in rats (*n* = 6) and the pharmacokinetic (PK) parameters are outlined in Table 5. The disparity between the two categories had a significant impact. All the IC formulations were better absorbed at the given dose compared to the free MTX powder. As shown in Figure 9, after 24 h of oral administration it was observed that the mean extent of absorption (C_max_) of all the IC formulations was substantially higher than that of free MTX (*p* < 0.05). Consistently, the relative bioavailability of MTX following oral administration of IC4 > IC1 > IC2 > IC3 was 2.20-, 1.84-, 1.74-, and 1.71-fold, respectively higher than that of free MTX. Furthermore, the T_max_, time needed to reach the C_max_ was short for IC4 formulation (T_max_ = 0.75 ± 0.29 h) and the elimination rate constant (K_el_) was slightly prolonged (0.15 ± 0.02 h^−1^). Therefore, the release and uptake of active MTX from the IC4 formulation occurred rapidly, with better bioavailability than those of the free MTX and other IC formulations implying that IC4 formulations had stronger absorption permitting rapid onset of action, and a longer retention in-vivo than the free drug. These findings are also consistent with in vitro dissolution and solubility studies, implying MTX/DM-β-CD IC formulation i.e., IC4, to be the more suitable oral formulation and DM-β-CD a better host molecule to encase lipophilic MTX. Complexation of MTX with CD molecules enhances the bioavailability of MTX by increasing the drug solubility, dissolution, and/or permeability, presenting it in a more soluble form at the absorption site, leading to its higher systemic absorption. Evidently, structural and functional changes to CDs will fine-tune the degree of complexation and, as a result, drug solubilization tendency and bioavailability benefits, suggesting that judicious CDs selection is crucial [48]. In terms of clinical implications, improved solubility and increased bioavailability achieved through the “host-guest” interaction of IC can enhance the systemic absorption of lipophilic molecules that inherently shows slow or erratic absorption profiles. In addition, it may enhance drug efficacy and potency, while lowering the dose-related toxicity by enabling them to be effective at lower dosages. Furthermore, these IC systems could be useful in reducing pharmaceutical formulation-related side effects by limiting the usage of high-concentration or large-number of excipients, lowering the drug-excipients interactions, and, therefore, displaying better tolerance and safety profile to the patients.

### 3.5. Photostability Study

**Figure 10:**
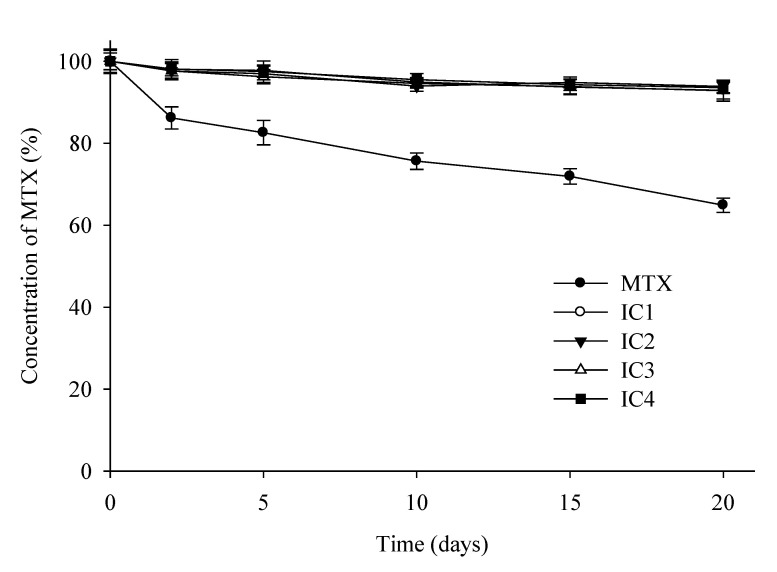
Residual concentration of MTX in inclusion compounds after exposure to forced photodegradation condition for 20 days. MTX/HP-β-CD inclusion complex (IC1); MTX/β-CD inclusion complex (IC2); MTX/M-β-CD inclusion complex (IC3); MTX/DM-β-CD inclusion complex (IC4). Each value represents the mean ± S.D. (*n* = 3).

The free MTX powder and the four IC formulations were subjected to UV irradiation and investigated for physical appearance, color, and drug content profiles. Figure 10 depicts the drug content profiles examined at different time intervals during the 20 days storage period. After being exposed to a UV light source, the free MTX powder was found to be significantly deteriorated, with a 36.8 ± 1.7% decrease in MTX concentration and distinctive color changes observed at the end of the study time. All the IC formulations, on the other hand, showed relatively minor photodegradation, with a 6.7 ± 1.9% drop in initial drug concentration, while the original pale-yellow color of MTX powder remained intact. These findings indicate that MTX-CD complexation results in the formation of stable ICs in which the photosensitive drug (guest) is entrapped inside the CD cavity (host) by a host-guest interaction, shielding the drug from direct exposure to a light source, and thereby preventing photolytic degradation.

### 3.6. Influence of DM-β-CD on Permeation Behavior of MTX

**Figure 11:**
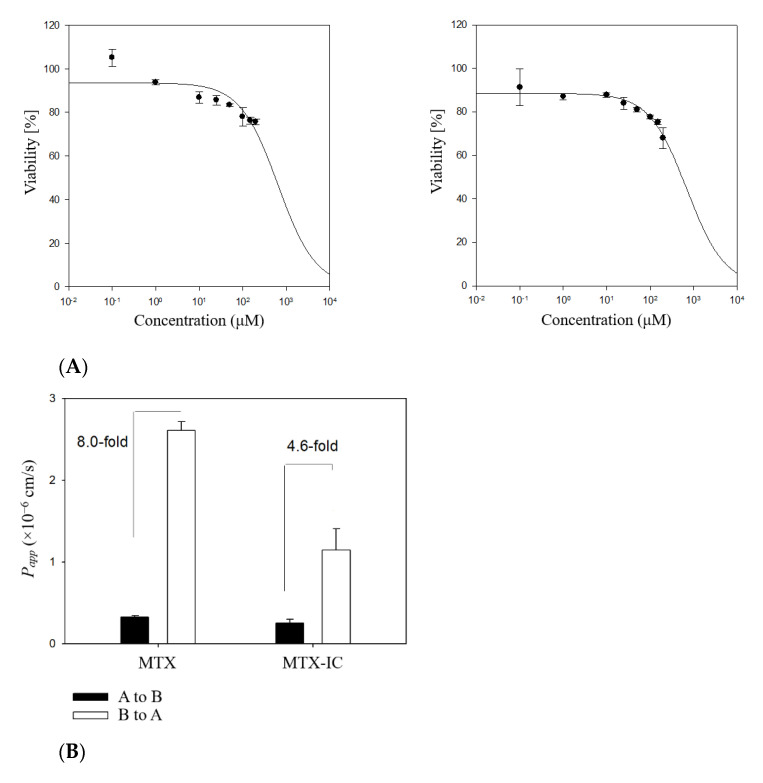
(**A**) Cytotoxic effect of MTX and the MTX/DM-*β*-CD inclusion complex (IC4) in Caco-2 cells (*n* = 6); and (**B**) the cumulative amount of MTX transported from A to B and B to A in Caco-2 cells.

To investigate the in-vitro permeability of MTX and MTX/DM-*β*-CD IC, cytotoxicity studies against Caco-2 cells were conducted by MTT assay, with the results given in Figure 11A. Up to 200 µM, no substantial cytotoxicity for MTX and MTX/DM-*β*-CD IC was observed. Cytotoxic agents such as doxorubicin and docetaxel have the half-maximal inhibitory concentration (IC_50_) values of 5 and 70 µM against Caco-2 cells [49,50]. Therefore, MTX and MTX-loaded IC are considered to have relatively weak cytotoxicity compared to common cytotoxic agents. In addition, the $P_{app}$ values of free MTX and IC were also compared, with results are shown in Figure 11B. The A to B transport represented by the $P_{app}$ values of MTX and MTX/DM-*β*-CD are comparable with 0.57 ± 0.14 × 10^−6^ cm/s and 0.65 ± 0.18 × 10^−6^ cm/s (*p* < 0.01), respectively. However, both samples’ B to A transport (efflux) was significantly higher than their influx rate (A to B transport), corroborating previous assertions that MTX is subjected to P-glycoprotein (P-gp) mediated efflux, resulting in lower systemic drug concentration. In particular, the efflux ratio (B to A/A to B) of MTX/DM-*β*-CD was substantially lower compared to the free MTX powder (4.6- vs. 8.0-fold). Hence, in addition to increased drug solubility due to the inclusion of MTX in DM-*β*-CD binary complexes, inhibition of the P-gp driven efflux system may have contributed to increased systemic absorption, therefore enhanced oral bioavailability of MTX. This is consistent with the prior findings, which found that DM-*β*-CD-based IC formulations effectively inhibit the P-gp efflux system of poorly water-soluble and low permeable molecules [28,51].

## 4. Conclusions

The goal of this study was to (i) investigate the saturation solubility of MTX in presence of β-CD and its derivatives, (ii) load MTX into different CDs cavity, (iii) protect MTX under photolytic conditions, and (iv) maximize the aqueous solubility, dissolution, in-vivo absorption, and intestinal permeability. In summary, MTX was embedded into CDs at 1:1 stoichiometry, resulting in MTX-CD inclusion complexes that were confirmed with several spectroscopic techniques. The SEM micrographs of all the spray-dried solid inclusion complexes revealed well-dispersed, sphere-shaped particles, while the DSC and XRD studies confirmed the transformation of inherent crystalline MTX to its amorphous state. Though all four inclusion complex formulations outperformed the free drug in terms of aqueous solubility, dissolution, stability, and oral bioavailability, the inclusion complex with DM-β-CD, in particular, had a stronger complexation ability to the drug, thus making it a better host molecule for MTX encapsulation. Furthermore, this study revealed that DM-β-CD influences the permeation of MTX through Caco-2 cells, and while the MTX’s absorptive permeability is unaltered, there were decreases in the P-gp mediated efflux of MTX when complexed with DM-β-CD. Therefore, the increase in MTX oral bioavailability is due to the collective effects of increased aqueous solubility, improved drug dissolution leading to higher absorption from the GI tract, and inhibition of the P-gp efflux mechanism. Overall, MTX/DM-β-CD is a straightforward and effective technique for improving the pharmacologically relevant properties of the poor water solubility of MTX.

## Figures and Tables

**Table 1:** Composition of various inclusion compounds; MTX/HP-β-CD inclusion complex (IC1); MTX/β-CD inclusion complex (IC2); MTX/M-β-CD inclusion complex (IC3); MTX/DM-β-CD inclusion complex (IC4).

Formulation	IC1	IC2	IC3	IC4
MTX (g)	1	1	1	1
Cyclodextrin (g)	3.08	2.50	2.71	2.93
0.01 M HCl ethanolic solution (mL)	200	200	200	200
Water (mL)	200	200	200	200

**Table 2:** Solubilization efficacy of MTX in different CD solution. Each value represents the mean ± SD (*n* = 3).

Cyclodextrins	Stability Constant (*K_α_*, M^−1^)
HP-*β*-CD	433.56
*β*-CD	552.25
M-*β*-CD	574.48
DM-*β*-CD	3114.69

**Table 3:** The particle size of methotrexate-loaded inclusion compounds; MTX/HP-β-CD inclusion complex (IC1); MTX/β-CD inclusion complex (IC2); MTX/M-β-CD inclusion complex (IC3); MTX/DM-β-CD inclusion complex (IC4).

	Dx (10), μm	Dx (50), μm	Dx (90), μm
MTX	3.60 ± 0.48	14.03 ± 2.97	103.73 ± 12.13
IC1	0.78 ± 0.15	3.56 ± 0.07	8.88 ± 0.29
IC2	1.20 ± 0.03	4.39 ± 0.12	13.35 ± 0.49
IC3	0.45 ± 0.04	3.26 ± 0.06	7.26 ± 0.32
IC4	0.38 ± 0.03	3.23 ± 0.05	7.65 ± 0.25

**Table 4:** Comparative ^1^H-NMR chemical shifts of (δ, ppm) studies of DM-β-CD, free MTX, MTX/DM-β-CD inclusion complex (IC), and their complexation induced shifts (Δδ). (Δδ ppm = δcomplex–δfree), ND: not detected.

H Protons	δ(DM-β-CD)	δ(IC)	Δδ (ppm)	H Protons	δ(MTX)	δ(IC)	Δδ (ppm)
H-1	4.97	4.97	-	H-7	8.57	8.59	0.02
H-2	3.70	3.70	-	H-9	4.78	4.79	0.01
H-3	3.20	3.26	+0.06	H-10	3.20	ND	ND
H-4	3.56	3.57	+0.01	-NH_2_(2)	7.51	7.92	0.39
H-5	3.34	3.36	+0.02	-NH_2_(4)	6.74	6.87	0.13
H-6	ND	ND	ND	H-13,15	7.73	7.73	-
OCH3	3.50	3.50	-	H-16,12	6.82	6.82	-
OCH3	3.25	3.25	-	H-18	8.19	8.19	-
				H-19	4.35	4.35	-
				H-21	2.05	2.06	0.01
				H-22	2.32	2.34	0.02

**Table 5:** Pharmacokinetic parameters of methotrexate (MTX)-loaded inclusion compounds; MTX/HP-β-CD inclusion complex (IC1); MTX/β-CD inclusion complex (IC2); MTX/M-β-CD inclusion complex (IC3); MTX/DM-β-CD inclusion complex (IC4).

Parameter	MTX	IC1	IC2	IC3	IC4
*AUC* (h ng/mL)	1738.71 ± 294.65	3198.39 ± 226.79 *	3016.90 ± 237.09 *	2973.33 ± 316.89 *	3820.27 ± 424.27 ^#^
*C*_max_ (ng/mL)	265.63 ± 57.05	567.93 ± 70.55 *	439.61 ± 155.24 *	561.44 ± 149.83 *	872.76 ± 62.19 ^#^
*T*_max_ (h)	1.13 ± 0.23	1.03 ± 0.25	1.57 ± 0.40	1.04 ± 0.21	0.75 ± 0.29
*t*_1/2_ (h)	5.40 ± 0.72	5.24 ± 0.99	5.29 ± 0.70	4.95 ± 0.30	4.82 ± 0.17
*K*_el_ (h^−1^)	0.13 ± 0.02	0.14 ± 0.03	0.13 ± 0.02	0.15 ± 0.02	0.15 ± 0.02

Each value represents the mean ± S.D. (*n* = 6). * *p* < 0.05 compared with MTX powder. ^#^ *p* < 0.05 compared with MTX powder and IC1-3.

## Data Availability

The data presented in this study are available in the paper or in the supplementary material here.

## References

[1] Bleyer W.A. (1978). The clinical pharmacology of methotrexate: New applications of an old drug. Cancer.

[2] Giletti A., Vital M., Lorenzo M., Cardozo P., Borelli G., Gabus R., Martínez L., Díaz L., Assar R., Rodriguez M.N. (2017). Methotrexate pharmacogenetics in Uruguayan adults with hematological malignant diseases. Eur. J. Pharm. Sci..

[3] Braun J., Rau R. (2009). An update on methotrexate. Curr. Opin. Rheumatol..

[4] Abolmaali S.S., Tamaddon A.M., Dinarvand R. (2013). A review of therapeutic challenges and achievements of methotrexate delivery systems for treatment of cancer and rheumatoid arthritis. Cancer Chemother. Pharmacol..

[5] Kim D.S., Cho J.H., Park J.H., Kim J.S., Song E.S., Kwon J., Giri B.R., Jin S.G., Kim K.S., Choi H.-G. (2019). Self-microemulsifying drug delivery system (SMEDDS) for improved oral delivery and photostability of methotrexate. Int. J. Nanomed..

[6] Chatterji D.C., Gallelli J.F. (1978). Thermal and photolytic decomposition of methotrexate in aqueous solutions. J. Pharm. Sci..

[7] Ray S., Joy M., Sa B., Ghosh S., Chakraborty J. (2015). pH dependent chemical stability and release of methotrexate from a novel nanoceramic carrier. RSC Adv..

[8] Santos A.C., Costa D., Ferreira L., Guerra C., Pereira-Silva M., Pereira I., Peixoto D., Ferreira N.R., Veiga F. (2021). Cyclodextrin-based delivery systems for in vivo-tested anticancer therapies. Drug Deliv. Transl. Res..

[9] Datta S., Grant D.J.W. (2004). Crystal structures of drugs: Advances in determination, prediction and engineering. Nat. Rev. Drug Discov..

[10] Giri B.R., Kwon J., Vo A.Q., Bhagurkar A.M., Bandari S., Kim D.W. (2021). Hot-melt extruded amorphous solid dispersion for solubility, stability, and bioavailability enhancement of telmisartan. Pharmaceuticals.

[11] Noh G., Keum T., Bashyal S., Seo J.-E., Shrawani L., Kim J.H., Lee S. (2022). Recent progress in hydrophobic ion-pairing and lipid-based drug delivery systems for enhanced oral delivery of biopharmaceuticals. J. Pharm. Investig..

[12] Sanches B.M.A., Ferreira E.I. (2019). Is prodrug design an approach to increase water solubility?. Int. J. Pharm..

[13] Kim J.S., Park H., Kang K.T., Ha E.S., Kim M.S., Hwang S.J. (2022). Micronization of a poorly water-soluble drug, fenofibrate, via supercritical-fluid-assisted spray-drying. J. Pharm. Investig..

[14] Kim D.-H., Lee S.-E., Pyo Y.-C., Tran P., Park J.-S. (2020). Solubility enhancement and application of cyclodextrins in local drug delivery. J. Pharm. Investig..

[15] Jansook P., Ogawa N., Loftsson T. (2018). Cyclodextrins: Structure, physicochemical properties and pharmaceutical applications. Int. J. Pharm..

[16] Bilensoy E., Bilensoy E. (2011). Cyclodextrins in Pharmaceutics, Cosmetics, and Biomedicine: Current and Future Industrial Applications.

[17] Soni S.S., Alsasa A., Rodell C.B. (2021). Applications of macrocyclic host molecules in immune modulation and therapeutic delivery. Front. Chem..

[18] Jicsinszky L., Cravotto G. (2020). Cyclodextrins in skin formulations and transdermal delivery. J. Ski. Stem Cell.

[19] Truzzi E., Rustichelli C., de Oliveira Junior E.R., Ferraro L., Maretti E., Graziani D., Botti G., Beggiato S., Iannuccelli V., Lima E.M. (2021). Nasal biocompatible powder of Geraniol oil complexed with cyclodextrins for neurodegenerative diseases: Physicochemical characterization and in vivo evidences of nose to brain delivery. J. Control. Release.

[20] Szente L., Singhal A., Domokos A., Song B. (2018). Cyclodextrins: Assessing the impact of cavity size, occupancy, and substitutions on cytotoxicity and cholesterol homeostasis. Molecules.

[21] Brewster M.E., Loftsson T. (2007). Cyclodextrins as pharmaceutical solubilizers. Adv. Drug Deliv. Rev..

[22] Przybyla M.A., Yilmaz G., Becer C.R. (2020). Natural cyclodextrins and their derivatives for polymer synthesis. Polym. Chem..

[23] Möller K., Macaulay B., Bein T. (2021). Curcumin encapsulated in crosslinked cyclodextrin nanoparticles enables immediate inhibition of cell growth and efficient killing of cancer cells. Nanomaterials.

[24] Gidwani B., Vyas A. (2015). A comprehensive review on cyclodextrin-based carriers for delivery of chemotherapeutic cytotoxic anticancer drugs. Biomed Res. Int..

[25] Connors K.A., Higuchi T. (1965). Phase solubility techniques. Adv. Anal. Chem. Instrum..

[26] Giri B.R., Kim J.S., Park J.H., Jin S.G., Kim K.S., Ud Din F., Choi H.G., Kim D.W. (2021). Improved bioavailability and high photostability of methotrexate by spray-dried surface-attached solid dispersion with an aqueous medium. Pharmaceutics.

[27] Bashyal S., Seo J.-E., Keum T., Noh G., Lamichhane S., Kim J.H., Kim C.H., Choi Y.W., Lee S. (2021). Facilitated buccal insulin delivery via hydrophobic ion-pairing approach: In vitro and ex vivo evaluation. Int. J. Nanomed..

[28] Giri B.R., Lee J., Lim D.Y., Kim D.W. (2021). Docetaxel/dimethyl-β-cyclodextrin inclusion complexes: Preparation, in vitro evaluation and physicochemical characterization. Drug Dev. Ind. Pharm..

[29] Caira M.R., Bourne S.A., Samsodien H., Smith V.J. (2015). Inclusion complexes of 2-methoxyestradiol with dimethylated and permethylated β-cyclodextrins: Models for cyclodextrin-steroid interaction. Beilstein J. Org. Chem..

[30] Fu Y., Kao W.J. (2010). Drug release kinetics and transport mechanisms of non-degradable and degradable polymeric delivery systems. Expert Opin. Drug Deliv..

[31] Carneiro S., Costa Duarte F., Heimfarth L., Siqueira Quintans J., Quintans-Júnior L., Veiga Júnior V., Neves de Lima Á. (2019). Cyclodextrin–drug inclusion complexes: In vivo and in vitro approaches. Int. J. Mol. Sci..

[32] Loftsson T. (2017). Drug solubilization by complexation. Int. J. Pharm..

[33] Kwon J., Giri B.R., Song E.S., Bae J., Lee J., Kim D.W. (2019). Spray-dried amorphous solid dispersions of atorvastatin calcium for improved supersaturation and oral bioavailability. Pharmaceutics.

[34] Singh A., Van den Mooter G. (2016). Spray drying formulation of amorphous solid dispersions. Adv. Drug Deliv. Rev..

[35] Periasamy R., Kothainayaki S., Sivakumar K. (2021). Host-guest inclusion complex of β-cyclodextrin and 4,4′-(1,4-phenylenediisopropylidene)bisaniline: Spectral, structural and molecular modeling studies. J. Mol. Struct..

[36] Geng Q., Li T., Wang X., Chu W., Cai M., Xie J., Ni H. (2019). The mechanism of bensulfuron-methyl complexation with β-cyclodextrin and 2-hydroxypropyl-β-cyclodextrin and effect on soil adsorption and bio-activity. Sci. Rep..

[37] Al-Marzouqi A.H., Shehatta I., Jobe B., Dowaidar A. (2006). Phase solubility and inclusion complex of itraconazole with β-cyclodextrin using supercritical carbon dioxide. J. Pharm. Sci..

[38] Manne A.S.N., Hegde A.R., Raut S.Y., Rao R.R., Kulkarni V.I., Mutalik S. (2020). Hot liquid extrusion assisted drug-cyclodextrin complexation: A novel continuous manufacturing method for solubility and bioavailability enhancement of drugs. Drug Deliv. Transl. Res..

[39] Dedroog S., Pas T., Vergauwen B., Huygens C., Van den Mooter G. (2020). Solid-state analysis of amorphous solid dispersions: Why DSC and XRPD may not be regarded as stand-alone techniques. J. Pharm. Biomed. Anal..

[40] Ghodke D., Ghodke G., Patil K., Nakhat P., Nakhat P., Naikwade N., Magdum C. (2010). Solid state characterization of domperidone: Hydroxypropyl-b-cyclodextrin inclusion complex. Indian J. Pharm. Sci..

[41] Gao S., Bie C., Ji Q., Ling H., Li C., Fu Y., Zhao L., Ye F. (2019). Preparation and characterization of cyanazine-hydroxypropyl-beta-cyclodextrin inclusion complex. RSC Adv..

[42] Sangwai M., Vavia P. (2013). Amorphous ternary cyclodextrin nanocomposites of telmisartan for oral drug delivery: Improved solubility and reduced pharmacokinetic variability. Int. J. Pharm..

[43] De Almeida Magalhães T.S.S., de Oliveira Macedo P.C., Kawashima Pacheco S.Y., da Silva S.S., Barbosa E.G., Pereira R.R., Costa R.M.R., Silva Junior J.O.C., da Silva Ferreira M.A., de Almeida J.C. (2020). Development and Evaluation of Antimicrobial and Modulatory Activity of Inclusion Complex of Euterpe oleracea Mart Oil and β-Cyclodextrin or HP-β-Cyclodextrin. Int. J. Mol. Sci..

[44] Martins M.H., Calderini A., Pessine F.B.T. (2012). Host-guest interactions between dapsone and β-cyclodextrin (Part II): Thermal analysis, spectroscopic characterization, and solubility studies. J. Incl. Phenom. Macrocycl. Chem..

[45] Yuan C., Jin Z., Xu X. (2012). Inclusion complex of astaxanthin with hydroxypropyl-β-cyclodextrin: UV, FTIR, 1H NMR and molecular modeling studies. Carbohydr. Polym..

[46] NE P., TV L., TA K., EO H., LD K. (2004). Inclusion complexes of carotenoids with cyclodextrins: 1H NMR, EPR, and optical studies. Free Radic. Biol. Med..

[47] Yang L.-J., Ma S.-X., Zhou S.-Y., Chen W., Yuan M.-W., Yin Y.-Q., Yang X.-D. (2013). Preparation and characterization of inclusion complexes of naringenin with β-cyclodextrin or its derivative. Carbohydr. Polym..

[48] Hemine K., Skwierawska A., Kleist C., Olewniczak M., Szwarc-Karabyka K., Wyrzykowski D., Mieszkowska A., Chojnacki J., Czub J., Nierzwicki L. (2020). Effect of chemical structure on complexation efficiency of aromatic drugs with cyclodextrins: The example of dibenzazepine derivatives. Carbohydr. Polym..

[49] Li H., Krstin S., Wang S., Wink M. (2018). Capsaicin and piperine can overcome multidrug resistance in cancer cells to doxorubicin. Molecules.

[50] Tran P.H.L., Wang T., Yang C., Tran T.T., Duan W. (2020). Development of conjugate-by-conjugate structured nanoparticles for oral delivery of docetaxel. Mater. Sci. Eng. C.

[51] Arima H., Yunomae K., Hirayama F., Uekama K. (2001). Contribution of P-glycoprotein to the enhancing effects of dimethyl-beta-cyclodextrin on oral bioavailability of tacrolimus. J. Pharmacol. Exp. Ther..

